# Comparison Analysis of Different DNA Extraction Methods on Suitability for Long-Read Metagenomic Nanopore Sequencing

**DOI:** 10.3389/fcimb.2022.919903

**Published:** 2022-06-28

**Authors:** Lei Zhang, Ting Chen, Ye Wang, Shengwei Zhang, Qingyu Lv, Decong Kong, Hua Jiang, Yuling Zheng, Yuhao Ren, Wenhua Huang, Peng Liu, Yongqiang Jiang

**Affiliations:** ^1^State Key Laboratory of Pathogen and Biosecurity, Beijing Institute of Microbiology and Epidemiology, Academy of Military Medical Sciences, Beijing, China; ^2^The Fifth Medical Center of PLA General Hospital, The Fifth School of Clinical Medicine, Anhui Medical University, Hefei, China; ^3^Department of Clinical Laboratory, Dongfang Hospital, Beijing University of Chinese Medicine, Beijing, China

**Keywords:** metagenomics, mechanical lysis, enzymatic lysis, pathogen diagnosis, microbiome

## Abstract

Metagenomic next-generation sequencing (mNGS) is a novel useful strategy that is increasingly used for pathogens detection in clinic. Some emerging mNGS technologies with long-read ability are useful to decrease sequencing time and increase diagnosed accuracy, which is of great significance in rapid pathogen diagnosis. Reliable DNA extraction is considered critical for the success of sequencing; hence, there is thus an urgent need of gentle DNA extraction method to get unbiased and more integrate DNA from all kinds of pathogens. In this study, we systematically compared three DNA extraction methods (enzymatic cell lysis based on MetaPolyzyme, mechanical cell lysis based on bead beating, and the control method without pre–cell lysis, respectively) by assessing DNA yield, integrity, and the microbial diversity based on long-read nanopore sequencing of urine samples with microbial infections. Compared with the control method, the enzymatic-based method increased the average length of microbial reads by a median of 2.1-fold [Inter Quartile Range (IQR), 1.7–2.5; maximum, 4.8) in 18 of the 20 samples and the mapped reads proportion of specific species by a median of 11.8-fold (Inter Quartile Range (IQR), 6.9–32.2; maximum, 79.27]. Moreover, it provided fully (20 of 20) consistent diagnosed results to the clinical culture and more representative microbial profiles (*P* < 0.05), which all strongly proves the excellent performance of enzymatic-based method in long-read mNGS–based pathogen identification and potential diseases diagnosis of microbiome related.

## Introduction

Metagenomic next-generation sequencing (mNGS) is a hypothesis-free and unbiased approach that has the potential to detect all the known and unidentified pathogens yet. Because of its target agnostic nature, mNGS enables the discovery of new organisms in clinical sample and is especially suitable for rare, novel, and atypical etiologies of complicated infectious diseases, as well as the molecular diagnosis of polymicrobial infections (Goldberg et al., 2015; Cummings et al., 2016; Gu et al., 2019). Although such an unbiased approach appears highly suitable for pathogen diagnosis, difference in pathogens lysis method results in different pathogen distribution (Mattei et al., 2019). Currently, the most common used method is mechanical lysis with hard bead-beating, which may result in excessive DNA fragmentation (Salonen et al., 2010). This method fades the advantage of long sequence reading for the emerging sequencing techniques such as Nanopore and PacBio (Rhoads and Au, 2015; Wang et al., 2021). Furthermore, longer sequence reads can increase taxonomic resolution of sequence classification because they are more readily classified to species or subspecies level; meanwhile, short reads are often difficult to classify to species accurately and can sometimes result in misdiagnoses (Schlaberg et al., 2017). Therefore, there is still an urgent need for optimized cell wall degradation methods that provide DNA with high integrity from all kinds of pathogens.

Urinary tract infections (UTIs) are one of the most common infections in human, which can be caused by the broader microorganisms of bacteria and fungi (Hasman et al., 2014; Zhang et al., 2022). The vast microbial diversity present results in different optimal DNA extraction methods for different cell wall structures and compositions (Maukonen et al., 2012). Therefore, urine metagenomic pathogen diagnosis studies require an optimized DNA extraction method ensuring efficient cell lysis, minimal DNA shearing and unbiased microbial DNA recovery. In addition, it also needs to generate the most representative distribution of present microbial species. Notably, urine can be collected non-invasively in large volumes and therefore represents an attractive target for diagnostic assays. Although there has been much attention and efforts paid on establishment of mNGS-based diagnosed assay for UTI (Imirzalioglu et al., 2008; Schmidt et al., 2017; Li et al., 2020), there has been fewer studies aimed to evaluate the compatibility of DNA extraction methods for emerging long-read mNGS testing.

In this study, we compared three DNA extraction methods of mechanical lysis, enzymatic lysis, and a control method (DNA extracted directly without pre–cell lysis). Using metagenomic nanopore sequencing as the indicator, we assessed the quantity and integrity of the extracted DNA, the microbial diversity recovery, and the proportion of target microbial reads while keeping all the other steps standardized, with the goal of selecting a most compatible DNA extraction method for greater identification of potential pathogens when using long-read mNGS–based pathogen diagnostic analysis.

## Materials and Methods

### Study Design

**Figure 1:**
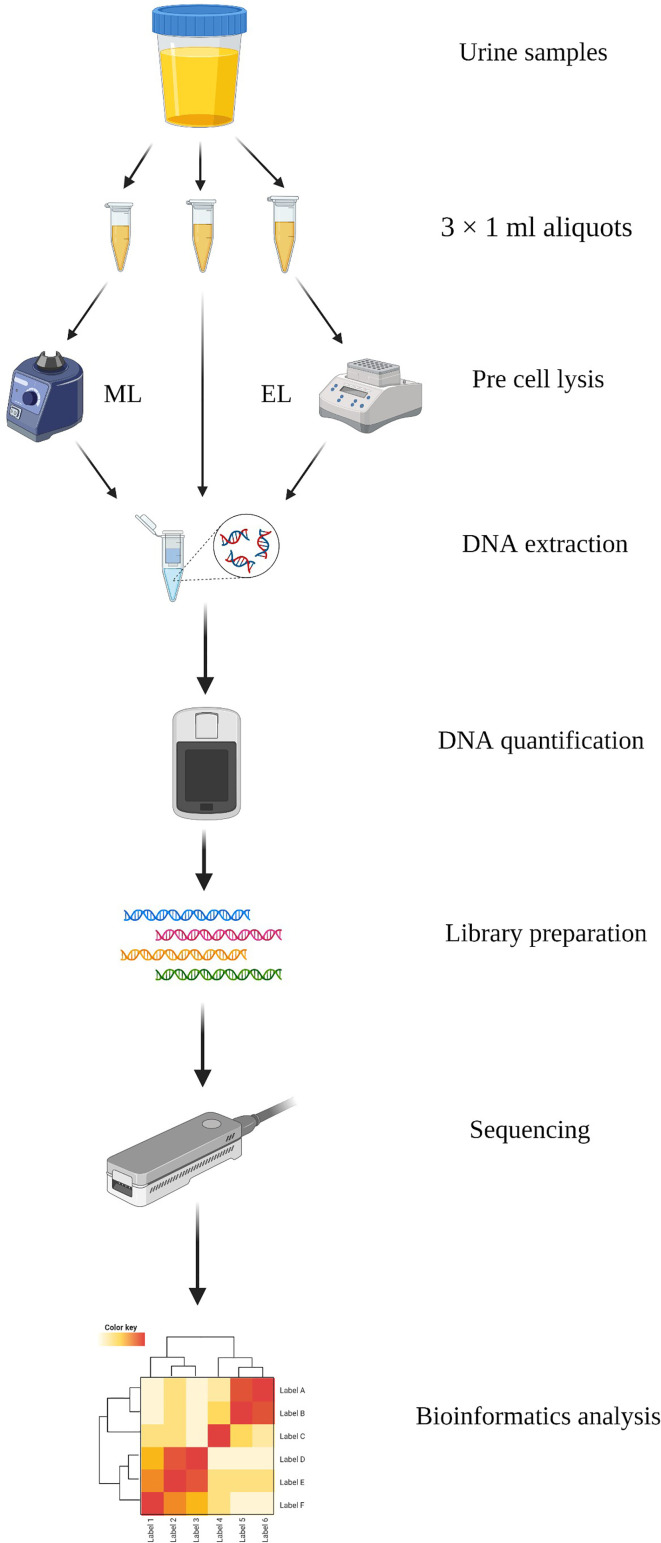
Schematic workflow of the study. DNA was extracted with three methods from urine samples and performed sequencing based on MinION. Created with BioRender.com.

DNA of the urine samples were extracted with three different methods in this study: Method 1, DNA extracted directly by the IndiSpin Pathogen Kit (Indical Bioscience); Method 2, DNA extracted based on mechanical lysis; Method 3, DNA extracted based on enzymatic lysis. We compared the three DNA extraction methods by evaluating the DNA yield and integrity, DNA recovery of specific species, and microbial diversity. Overview of this study is shown in **Figure 1**.

### Subjects and Urine Sample Collection

A clinical diagnosis of UTI required to refer to the culture result and consider indicators including a white blood cell count of > 10^7^/L, an epithelial cell count of < 10^7^/L, fever, dysuria, frequency of urination, and urgency (Willner et al., 2014; Kumar et al., 2015). In addition, the following criteria were used to determine inclusion in this study: the patients who had a few symptoms including urinary urgency, frequent urination, and painful urination; and the culture results were available and positive. Urine samples with less than 1 ml remained or with more than three species positive in culturation were excluded. There were 20 urine samples finally collected from 20 adults included in this study. Immediately after collection, samples were transported on ice and stored at −80°C prior to DNA extraction by three different methods. This study was approved by the Institutional Review Board (IRB) of the Beijing Dongfang Hospital (reference no. JDF-IRB-2020003101). All samples were obtained with the patient’s consent.

### DNA Extraction Methods

Each urine sample was aliquoted (1 ml) into three 1.5-ml Eppendorf tubes (Eppendorf) and centrifuged at 20,000 × g for 5 min to enrich for microbes. Then, 800 μl of supernatant was discarded, and the pellet was resuspended in the residual volume (200 µl) by gentle vortex to prepare the enriched urine samples. The detailed methods to extract DNA are listed below.

#### (i) Method 1. DNA Extraction Directly Without Pre–Cell Lysis

One aliquot of each enriched urine samples (200 μl) was used to extract DNA directly by the IndiSpin Pathogen Kit without pre–cell lysis, to be a method control. Briefly, after 200 μl of urine sample was added to a 20-μl aliquot of Proteinase K, 100 μl of Buffer VXL including 1 μg of Carrier RNA was added to the mixture and incubated for 15 min at 20°C–25°C. After this, 350 μl of Buffer ACB was added to the samples and mixed thoroughly by pulse vortex. Then, all the lysates were transferred to the Mini column and centrifuged at 6,000 × g for 1 min. The collection tubes containing the filtrate were discarded and placed the Mini column in the clean collection tubes. Six hundred microliters of Buffer AW1 was added to the Minin column for washing the DNA by a centrifugation of 6,000 × g for 1 min. The washing step above was repeated using 600 μl of Buffer AW2. After this, the membrane was dried by centrifuging at 20,000 × g for 2 min with clean collection tubes. Finally, the DNA was eluted by 100 μl of Buffer AVE. The concentrations of DNA were measured using Qubit 4.0 fluorometer with the dsDNA HS Assay kit (Thermo Fisher Scientific).

#### (ii) Method 2. DNA Extraction With Mechanical Lysis

Mechanical lysis of cell walls was accomplished with bead beating. One aliquot of enriched urine samples (200 μl) was transferred into Pathogen Lysis Tubes (Qiagen) with glass beads, and 50 μl of Buffer ATL (containing Reagent DX, Qiagen) was added according to the manufacturer’s instructions. The Pathogen Lysis Tubes were then attached to a horizontal platform on a vortex mixer and vortexed for 10 min at maximum speed. After that, the Pathogen Lysis Tubes were removed and briefly spined to collect any drops from the inside of the lid. DNA was extracted from the supernatant using the IndiSpin Pathogen Kit as described in Method 1.

#### (iii) Method 3. DNA Extraction With Enzymatic Lysis

One aliquot of enriched urine samples (200 μl) was used to extract DNA by enzymatic lysis method. Five microliters of lytic enzyme solution (Qiagen) and 10 µl of MetaPolyzyme [Sigma Aldrich; reconstituted in 750 µl of Phosphate Buffer Saline (PBS)] were added to the 200-µl samples and mixed by gentle pipetting. Mixed samples were incubated at 37°C in shaker for 1 h to lyse microbial cells. DNA was extracted from each post-lysed sample using the IndiSpin Pathogen Kit as described in Method 1.

### Library Preparation and Sequencing

All the samples mNGS testing were based on MinION platform (Oxford Nanopore Technology (ONT)). The samples included in this data set were processed and sequenced regardless of microbial DNA concentration to provide an accurate representation of the data that would likely be obtained from metagenomic analysis of urine in clinical settings.

Library preparation was performed using the PCR Barcoding Kit (SQK-PBK004, ONT) according to the manufacturer’s instruction, with 2-min extension and 15 cycles in the PCR amplification step. Up to six barcoded samples were loaded per flow cell for each sequencing run. Full details regarding library preparation are provided in **Supplementary Methods**.

Nanopore sequencing was performed using R9.4.1 flow cells (FLO-MIN106) on MinION. A total of 75 µl of library DNA was loaded into the flow cell according to the manufacturer’s instructions. ONT MinKNOW GUI software (version 4.2.8) was used to collect raw sequencing data.

### Bioinformatic Analysis

The raw sequencing data were processed using our automatic bioinformatics pipeline composed of a set of fixed external software (ont-Guppy, bwa, SAMtools, BLASTn). The processing step consists of (1) trimming adapters using ont-Guppy; (2) subtraction human host sequences mapped to the human reference genome (GRCh38, https://www.ncbi.nlm.nih.gov/data-hub/assembly/GCF_000001405.39/) using Burrows–Wheeler alignment with BWA-MEM algorithm; (3) output SAM file was indexed and sorted with SAMtools (version 1.7) to generate nonhuman reads; (4) all the nonhuman reads were classified by simultaneous alignment to RefSeq microbial genome databases (ftp://ftp.ncbi.nlm.nih.gov/genomes/refseq) consisting of viruses, bacteria, fungi, and parasites using BLASTn (version 2.10.1); (5) species classification result was finally outputted as.csv file after processing by two custom Python scripts and Linux commands. The automatic bioinformatics pipeline is available at https://github.com/gitzl222/APDNS/.

### Statistical Analysis

All statistical analyses were performed using GraphPad Prism version 8.4. Normality was tested for all datasets using the D’Agostino Pearson omnibus normality test, and correlation was analyzed using Pearson correlation. All data were log-transformed and further analyzed using Kruskal–Wallis test or the two-tailed paired t-test as appropriate to calculate the statistical significance between the methods. A *P*-value less than 0.05 was considered as statistically significant.

## Results

### DNA Yield and Integrity

**Figure 2:**
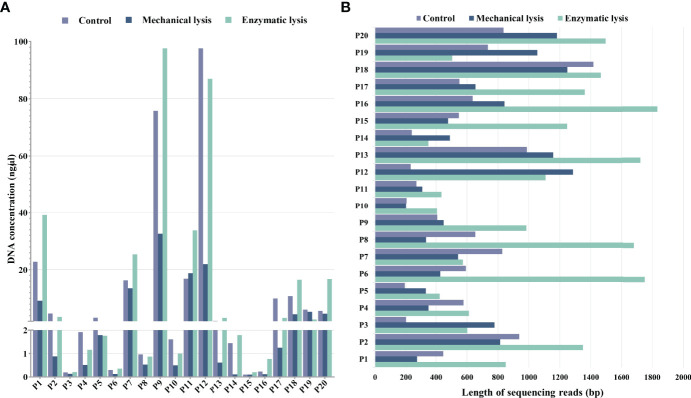
DNA yield and integrity extracted using mechanical lysis and enzymatic lysis. **(A)** DNA concentrations extracted by three different methods. **(B)** The absolute average length of microbial sequencing reads.

The purpose of this study was to select and statistically validate an optimal method for the microbial DNA extraction to be applied in long-read mNGS–based pathogen diagnosis of clinical samples. Three extraction methods were compared using clinical urine samples, and their DNA yield, integrity, and the specific species abundance were used as screening criteria to determine the best method. For differences analysis of DNA yield and integrity between the three DNA extraction methods, we counted the DNA concentrations and the average length of microbial reads and found that they varied a lot not only between samples of each method but also between different DNA extraction methods (**Figure 2**). We further investigated the statistical difference among them.

**Figure 3:**
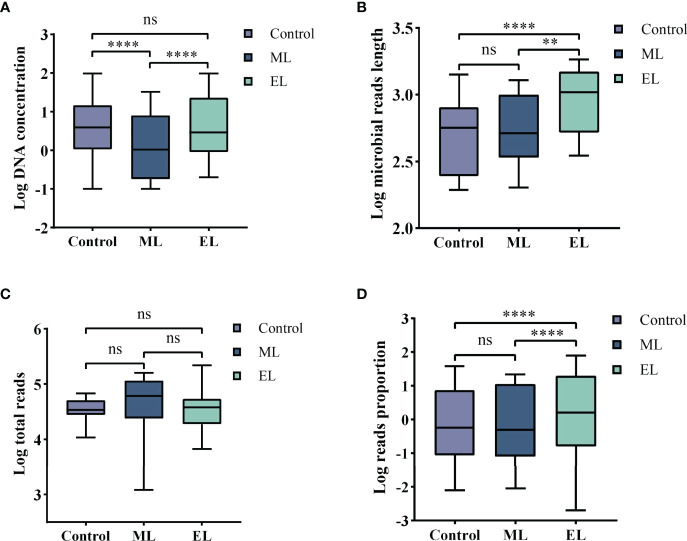
Boxplots demonstrating the statistic difference analysis for the three DNA extraction methods. Median values are indicated by the line within the boxplot. The box extends from the 25th to 75th percentiles, and whiskers indicate the minimum and maximum values. ****P < 0.0001; **P < 0.01; ns, nonsignificant. **(A)** Log DNA concentration. **(B)** Log average length of microbial reads. **(C)** Log total reads. **(D)** Log mapped reads proportion of the specific species.

For DNA yield, we found that DNA concentrations extracted by mechanical-based method are significantly lower than the control method (*P* < 0.0001, **Figure 3A**), whereas the enzymatic-based method showed no significant differences with control method (*P* > 0.05, **Figure 3A**). This result indicated that enzymatic-based method has no extra effect, but mechanical lysis with bead-beating has negative effects on DNA yield.

For DNA integrity, we found that the average length of microbial reads generated by enzymatic-based method was significantly longer than the control method (*P* < 0.0001, **Figure 3B**) and mechanical-based method (*P* < 0.01, **Figure 3B**), increased by a median of 2.1-fold (IQR, 1.7–2.5; maximum, 4.8) in 18 of the 20 samples and 1.9-fold (IQR, 1.4–2.3; maximum, 5.0) in 16 of the 20 samples (**Table 1**), respectively.

**Table 1:** DNA yield and integrity of the three DNA extraction methods.

SP	Control		ML Method		EL Method	
	Yield(ng/μl)	Length(bp)	Yield(ng/μl)	Length(bp)	Yield(ng/μl)	Length(bp)
P1	22.8	446.2	9.2	273.2	39.2	849.7
P2	4.68	936.9	0.878	814.5	3.5	1348.7
P3	0.198	201.9	0.128	777.7	0.204	602.2
P4	1.92	577.8	0.51	350.9	1.16	612.3
P5	3.28	193.5	1.79	333.6	1.76	423.4
P6	0.294	592	0.126	427.3	0.352	1752.8
P7	16.3	828.7	13.5	541.9	25.4	573.3
P8	0.966	653.8	0.532	334.9	0.872	1682.5
P9	75.8	407.1	32.6	449.1	97.6	983.8
P10	1.61	205.2	0.496	201.5	1	406
P11	16.9	268.3	18.8	311	33.8	435
P12	97.6	231.4	22	1284.1	87	1107.6
P13	2.18	987.2	0.612	1157.1	3.24	1723.6
P14	1.45	238.6	0.11	489.6	1.79	349.6
P15	0.1	547.5	0.1	477.1	0.2	1248
P16	0.232	636.2	0.116	842.2	0.77	1834
P17	10	551.3	1.25	654.9	3.18	1361.3
P18	10.8	1416.9	4.46	1249.4	16.5	1465.5
P19	6.08	735	5.32	1054.9	2.66	503.9
P20	5.64	836.5	4.64	1181.4	16.8	1495.9

SP, sample; ML, mechanical lysis; EL, enzymatic lysis.

### Abundance Variation for Specific Species

To determine adaption of these three DNA extraction methods for mNGS-based pathogen diagnosis, we counted the consistency between results of culture and mNGS and found that the enzymatic-based method provided a fully consistent result while the other two methods gave 15 of 20 and 14 of 20, respectively (**Table 2**). To evaluate the abundance variation of specific species, we next calculated the mapped reads number and proportion of the specific species that can be identified by culture (**Table 2****)**. By calculating the sequencing depth of all three DNA extraction methods, we found that the total number of reads generated by MinION of each sample showed no significant difference among all the three DNA extraction methods (all *P* > 0.05, **Figure 3C**). On the basis of the same depth of sequencing, the enzymatic-based method increases the mapped reads proportion of specific species by a median of 11.8-fold (IQR, 6.9–32.2; maximum, 79.27; **Figure 3D**) in 14 of the 20 samples compared with the control method, except one from a gram-negative bacteria *E. coli* infection (P19). In particular, the remaining five samples (P3, P5, P7, P10, and P14) were found of significant increase in reads number of specific species from “no reads detected” to “large number of reads detected”. The mechanical-based method showed a decreased proportion of mapped reads in most (9 of 15) samples although no significant difference observed compared with the control method. Furthermore, the five samples with no reads of specific species detected in the control method were also not detected any targeted reads in mechanical-based DNA extraction method. Finally, sample P1, which could be correctly detected by control method, was not detected by mechanical-based method, indicating that this method may lead to loss of microbial DNA sequences.

**Table 2:** Abundance variation of specific species by different DNA extraction methods.

SP	Cultureresult	Control		ML Method		EL Method	
		Num	Pro	Num	Pro	Num	Pro
P1	*Candida albicans*	11	0.102%	0	0%	127	1.16%
P2	*Candida glabrata*	25	0.201%	70	0.099%	957	1.32%
P3	*Candida glabrata*	0	0%	0	0%	117	0.47%
P4	*Candida parapsilosis*	174	0.533%	415	0.26%	57069	31.4%
P5	*Candida glabrata*	0	0%	0	0%	32	0.077%
P6	*Candida glabrata*	1202	2.945%	201	1.430%	4212	23.2%
P7	*Candida albicans*	0	0%	0	0%	42	0.02%
P8	*Candida parapsilosis*	116	0.234%	179	0.14%	713	4.07%
P9	*Candida albicans*	2	0.008%	3	0.013%	118	0.27%
P10	*Candida albicans*	0	0%	0	0%	1	0.002%
P11	*Candida albicans*	3	0.008%	11	0.009%	20	0.094%
P12	*Candida glabrata*	3	0.024%	12	0.057%	638	1.39%
P13	*Enterococcus Faecium*	7222	16.52%	12305	22%	15516	45.1%
	*Candida albicans*	268	0.613%	281	0.5%	4966	14.4%
P14	*Trichosporon asahii*	0	0%	0	0%	53	0.093%
P15	*Candida krusei*	11910	38.571%	252	20.8%	44865	79.1%
P16	*Candida parapsilosis*	1234	2.164%	106	5.8%	17235	59.84%
P17	*Candida albicans*	56	0.082%	36	0.08%	2774	6.5%
P18	*Klebsiella pneumoniae*	3097	10.231%	15168	11.3%	1048	15.6%
P19	*Escherichia coli*	12553	22.157%	5385	20.1%	1503	17.2%
P20	*Candida glabrata*	323	1.139%	429	1.27%	437	1.61%
*Con*		15/20		14/20		20/20	

SP, sample; Num, number; Pro, proportion; Con, consistency.

### Impact of DNA Extraction Method on Microbial Diversity Composition

**Figure 4:**
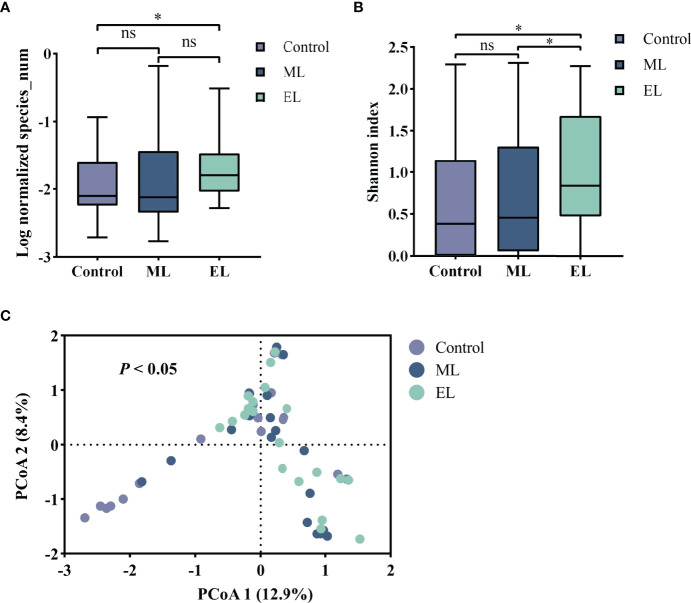
The microbial diversity of the three DNA extraction methods. Median values are indicated by the line within the boxplot. The box extends from the 25th to 75th percentiles, and whiskers indicate the minimum and maximum values. *P < 0.05; ns, nonsignificant. **(A)** Log normalized total number of microbial species observed by mNGS. **(B)** Alpha diversity based on the Shannon index. **(C)** Beta diversity with PCoA based on the Bray–Curtis index.

To evaluate the impact of DNA extraction method on microbial diversity composition, we quantified the relative abundance of microbial taxa per sample based on nanopore sequencing. We first compared the total number of microbial species for each of the three DNA extraction methods (**Figure 4A**). The total number of microbial species was normalized by the total number of reads per sample and made pairwise comparison across the three DNA extraction methods (see **Supplementary Table S1** for raw data). Enzymatic-based method observed more microbial species in urine samples than the control method (*P* < 0.05), whereas the other two methods gave no significant difference of microbial species diversity (*P* > 0.05). We further evaluated the microbial diversity variation by the alpha and beta diversities. Alpha diversity by Shannon index indicated that significant increase of microbial diversity was observed in enzymatic-based method compared with the other two methods (all *P* < 0.05, **Figure 4B**). The beta diversity with principal coordinate analysis (PCoA) was based on the Bray–Curtis dissimilarity, and the PERMANOVA test showed a significant difference of the microbial composition among these three methods (*P* < 0.05, **Figure 4C**).

**Figure 5:**
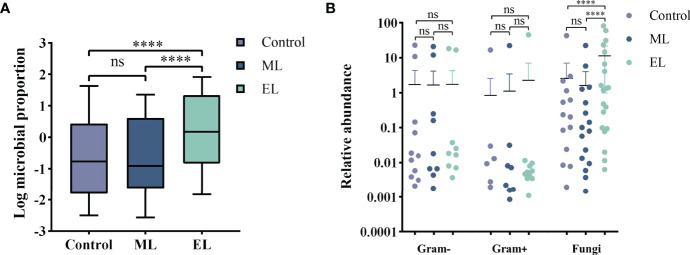
Evaluation of the DNA extraction efficiency for the three DNA extraction methods. ****P < 0.0001; ns, nonsignificant. **(A)** Boxplot of Log proportion of microbial reads in total reads generated by MinION. Median values are indicated by the line within the boxplot. The box extends from the 25th to 75th percentiles, and whiskers indicate the minimum and maximum values. **(B)** Relative abundance difference for different types of species by different DNA extraction methods.

**Figure 6:**
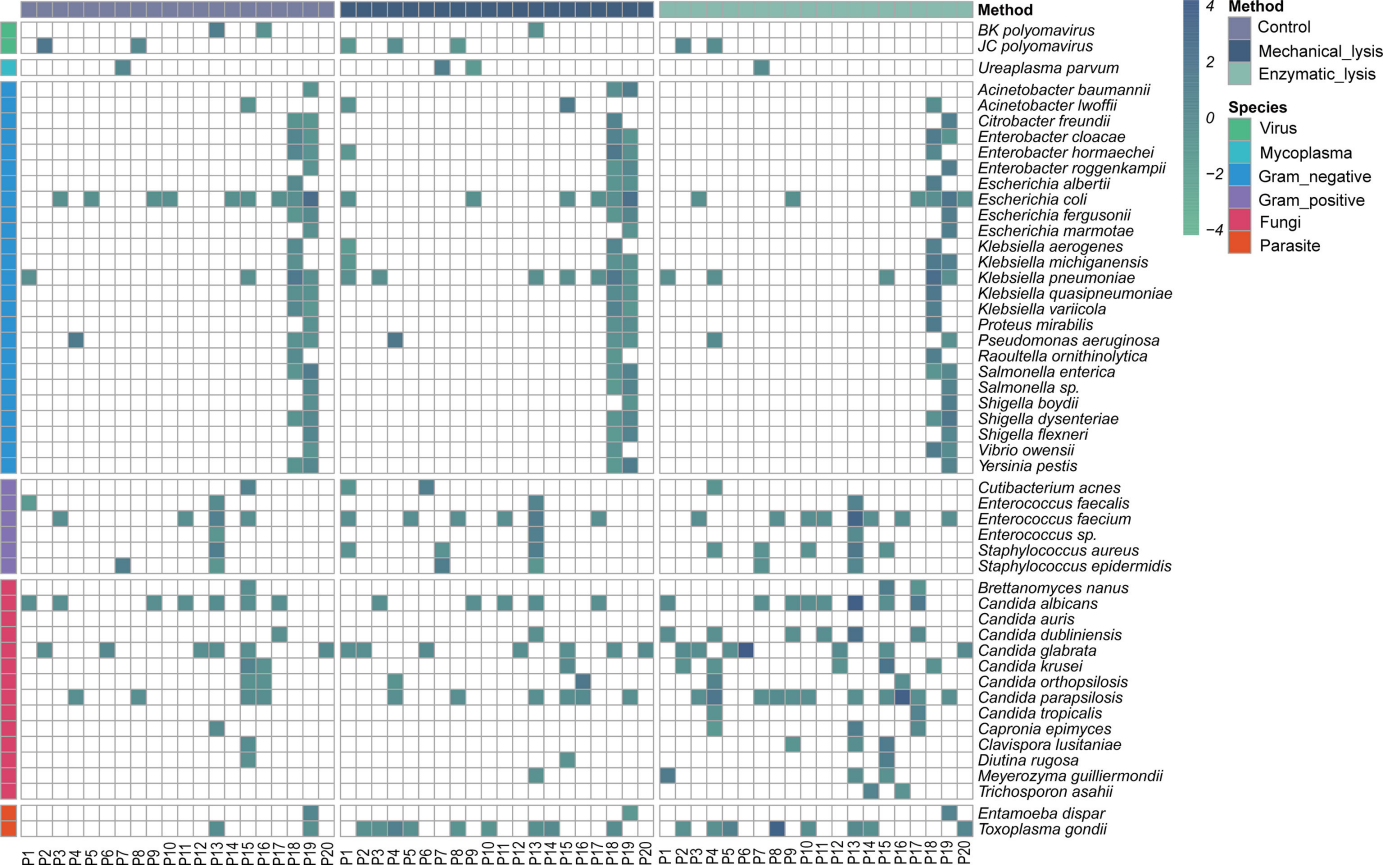
Distribution and relative abundance of microbial profile in urine samples with DNA extracted by different methods. Heatmap strip at the left and top are to aid in distinguishing different species types and DNA extraction methods, respectively.

For evaluating the microbial DNA extraction efficiency ratio of the three methods, we compared the proportion of total microbial reads per sample for each method (see **Supplementary Table S1** for raw data). Similarly, enzymatic-based method increased the microbial proportion by a median of 9.2-fold (IQR, 3.1–26.0; maximum, 69.0; **Figure 5A**) compared with control group, whereas the mechanical-based method had a median of 0.9-fold (IQR, 0.6–1.2; maximum, 3.3; **Figure 5A**). In addition, compared with the mechanical-based method, enzymatic-based method increased the microbial proportion by a median of 11.9-fold (IQR, 3.3–22.1; maximum, 74.5; **Figure 5A**). To assess which types of species were most impacted by the extraction methods, we investigated the distribution and relative abundance of the most common species (**Figures 5**, **6**). We found that gram-positives had a visible variation and fungi species had a significant variation (*P* < 0.0001, **Figure 5B**) in relative abundance across methods, whereas the variation in gram-negatives abundance was not obvious. These results are in line with previous observations that gram-positive bacteria and fungi are more likely to be affected by DNA extraction methods (McOrist et al., 2002; Santiago et al., 2014; Ackerman et al., 2019). In addition, these results also showed low bacterial abundance in samples from fungal infected patients. Likewise, in samples from bacteria-infected patients (P18 and P19), the fungal abundance was low. We further compared the microbial relative abundance of these three methods using Kruskal–Wallis test and found that significant difference existed between each other (all *P* < 0.0001).

## Discussion

In metagenomic sequencing studies, variations in the DNA extraction protocol can have important downstream effects on the observed microbial composition. Maximizing DNA concentration while also minimizing fragmentation are key aspects to consider when selecting an extraction method. This is both because high-quality libraries are required for sequencing, and protocols that consistently recover low-yield or highly fragmented DNA are likely to skew the observed community composition (Costea et al., 2017). The emergence of new long-read sequencing techniques such as nanopore has raised the bar for DNA quality and extraction methods. However, there is a paucity of studies to evaluate performance of different DNA extraction methods for long-read mNGS–based pathogen diagnostic testing. In this study, for selecting a best suited DNA extraction method to support pathogen diagnosis based on long-read mNGS testing, we systematically compared three DNA extraction methods by assessing DNA yield, integrity, and the microbial diversity based on metagenomic nanopore sequencing of urine samples from patients with UTI.

Among the three methods, the DNA concentration of the mechanical-based method was significantly lower compared with the other methods, which may be result from the loss of excessive short DNA sequences during DNA purification on silica membrane (Dilley et al., 2021). However, we performed analysis of correlation to compare DNA yield and microbial proportion within each DNA extraction method, and there were no correlations observed (all *P* > 0.1) as host DNA accounts for a large proportion in urine samples. These results are in line with published literature (Yuan et al., 2012). Therefore, DNA yield alone appears to be an unrepresentative measure for extraction efficiency because microbial DNA accounts for a little proportion of total DNA in urine samples (Salonen et al., 2010).

For the comparison of microbial reads length generated by mNGS testing, although there was no significant difference observed between mechanical-based method and control method, enzymatic-based method generated much longer-read length, indicating that the long DNA sequences had been released to a greater extent after the enzymatic cell lysis and resulting in outputted DNA with high integrity. Hence, these results proved the better compatibility of enzymatic-based method to the long-read sequencing technologies. Unusually, this result also seems to show that mechanical-based method did not make excessive shearing of DNA. However, it can also be interpreted with the preference of the silica membrane to capture longer DNA sequences (Doran and Foran, 2014; Dilley et al., 2021), which is also in line with the result of lower DNA yield of mechanical-based method above.

Pathogen diagnosis using MinION-based mNGS testing is common in recent studies (Schmidt et al., 2017; Charalampous et al., 2019; Moon et al., 2019). Unbiased cell lysis and complete DNA extraction of all microbial pathogens are crucial to recover specific pathogenic species accurately in mNGS testing, as reads derived from the normal microbiota in human may influence pathogens identification (Gu et al., 2019). Among the 20 urine samples with microbial infection, enzymatic-based mNGS testing provided a fully consistent result with pathogen detected by culture. In contrast, control method and mechanical-based method missed detection of five and six samples, respectively. All the missed pathogens were fungal pathogens, indicating that some fungal cells are more difficult to lyse, again, in line with the result from a previous study (Ackerman et al., 2019). In addition, on the basis of the detection result of control method and enzymatic method, the cell lysis treatment in advance is necessary and effective for DNA extraction of clinical samples with unknown infectious agents when using sequence-based detection methods, although it increases the turnround time of DNA extraction.

The microorganisms that colonize various anatomical sites of the human body play important roles in human health and disease (Dethlefsen et al., 2007), it is critical to understand the urinary microbiome comprehensively and accurately to develop novel therapies for UTI (Xiao et al., 2016; Neugent et al., 2020). Enzymatic-based method provided the largest normalized species number and the microbial proportion among the three methods; especially for the recovery of fungi and gram-positive microbiota, the enzymatic-based method obtained a most high abundance, indicating that it can generate a more representative microbial diversity composition from urine samples. These results proved that the enzymatic-based method can serve as an unbiased and reliable procedure for DNA extraction in the future sequence-based metagenomic analyses.

This study presents some limitations. First, it is difficult to assess which DNA extraction method came closest to the biological truth for absence of parallel evaluation of DNA extraction methods with a mock community. Second, we did not investigate any additional factors that may affect the metagenomic results, such as that of reagent and laboratory contamination (Salter et al., 2014).

In conclusion, we proved excellent performance of enzymatic-based method for long-read mNGS testing through systematically comparing three DNA extraction methods. We anticipate that procedures for DNA extraction will likely further improve in the future and propose that using a combination of lytic enzyme solution and MetaPolyzyme for effective lysis of a range of microbes, including both fungi and bacteria with minimal shearing. Although we have only proved the advantage of enzymatic-based DNA extraction method on urine samples, this can probably be extended to other samples such as stool and bronchoalveolar lavage fluid. By combining reliable organism lysis, unbiased sequencing, and comprehensive reference databases, long-read mNGS testing can be applied in real clinical practice for hypothesis-free and universal pathogen detection, promising to improve diagnostic accuracy of all microbiological infections.

## Data Availability Statement

The datasets presented in this study can be found in online repositories. The names of the repository/repositories and accession number(s) can be found in the article/**Supplementary Material**.

## Ethics Statement

The studies involving human participants were reviewed and approved by the Institutional Review Board (IRB) of the Beijing Dongfang Hospital (reference no. JDF-IRB-2020003101). The patients/participants provided their written informed consent to participate in this study.

## Author Contributions

WH, PL, YJ, and LZ conceived and designed the study. LZ and TC carried out the experimental work and analyzed the data. LZ, TC, and YW conceptualized the experimental methods, performed bioinformatics, and wrote the original draft of the manuscript. PL, WH, and YJ participated in the review and editing of the manuscript. All authors contributed to the article and approved the submitted version.

## Funding

The project was financed by the National Natural Science Foundation of China (82002115).

## Conflict of Interest

The authors declare that the research was conducted in the absence of any commercial or financial relationships that could be construed as a potential conflict of interest.

## Publisher’s Note

All claims expressed in this article are solely those of the authors and do not necessarily represent those of their affiliated organizations, or those of the publisher, the editors and the reviewers. Any product that may be evaluated in this article, or claim that may be made by its manufacturer, is not guaranteed or endorsed by the publisher.
